# Gamma irradiation induced surface modification of (PVC/HDPE)/ZnO nanocomposite for enhancing the oil removal and conductivity using COMSOL multiphysics

**DOI:** 10.1038/s41598-023-34583-0

**Published:** 2023-05-09

**Authors:** Mohamed Mohamady Ghobashy, Amal. F. Abd El-Gawad, S. A. Fayek, M. A. Farahat, M. I. Ismail, Ahmed M. Elbarbary, A. I. Sharshir

**Affiliations:** 1 Radiation Research of Polymer Chemistry Department, National Center for Radiation Research and Technology (NCRRT), Egyptian Atomic Energy Authority (EAEA), Cairo, Egypt; 2 Faculty of Engineering, Zagazig University, Zagazig, Egypt; 3 Solid State and Accelerator Department, National Center for Radiation Research and Technology (NCRRT), Egyptian Atomic Energy Authority (EAEA), Cairo, Egypt; 4 Faculty of Computers and Informatics, University Zagazig, Zagazig, Egypt; 5 Faculty of Engineering, Egypt University of Informatics, Cairo, Egypt

**Keywords:** Chemistry, Energy science and technology, Engineering, Materials science, Mathematics and computing, Nanoscience and technology, Optics and photonics, Physics

## Abstract

Blend nanocomposite film was prepared by loadings of irradiated ZnO in ratios of (5 wt%) inside the PVC/HDPE matrix using a hot-melt extruder technique. The physical and chemical properties of the irradiated and unirradiated ZnO samples are compared. The Vis–UV spectrum of ZnO shows an absorption peak at a wavelength of 373 nm that was slightly red-shifted to 375 nm for an irradiated sample of ZnO at a dose of 25 kGy due to the defect of crystal structure by the oxygen vacancy during gamma irradiations. This growth of the defect site leads to a decrease in energy gaps from 3.8 to 2.08 eV. AC conductivity of ZnO sample increased after the gamma irradiation process (25 kGy). The (PVC/HDPE)/ZnO nanocomposites were re-irradiated with γ rays at 25 kGy in the presence of four different media (silicon oil, sodium silicate, paraffin wax and water). FTIR and XRD were performed to monitor the changes in chemical composition. The new peak at 1723 cm^−1^ attributed to C=O groups was observed in irradiated (PVC/HDPE)ZnO samples at only sodium silicate and water media. This process induced new function groups on the surface of the (PVC/HDPE)/ZnO blend sample. This work aims to develop (PVC/HDPE)ZnO for oil/water separation. The highest oil adsorption capability was observed in samples functionalized by C=O groups based on the different tested oils. The results suggest that the surface characterization of the (PVC/HDPE)/ZnO can be modified to enhance the oil adsorption potential. Further, the gamma irradiation dose significantly enhanced the AC conductivity compared to the unirradiated sample. According to COMSOL Multiphysics, the irradiated sample (PVC/HDPE)ZnO in water shows perfect uniform electric field distribution in medium voltage cables (22.000 V).

## Introduction

The production of polymers with specified physical–chemical properties combined with the features granted by surface modifications has become possible through the fascinating and practical surface modification of polymeric materials^1–4^. There are various ways to change the properties of polymers, including blending, grafting, and curing. The physical blending of two (or more) polymers results in the desired characteristics. In the process known as “grafting”, monomers are covalently attached (modified) to the polymer chain. In contrast, an oligomer mixture is polymerized during curing to generate a coating physically attached to the substrate. Grafting is a promising approach for adding unique functional groups to polymers to change their original properties and expand the range of their applications^5,6^.

After the irradiation process some atoms and groups such as hydrogen atoms and carbon-hydrogen groups are released from polymers, there is a considerable shift in the stoichiometry of the polymer. If present in the polymer chains, other atomic species (O, F, Cl, N, etc.) are also expelled^7–9^. It is well known that after radiation exposure, polymers lose hydrogen, which has the effect of physical polymer properties. Chain scission produces smaller units and oligomer chain, a profusion of double bonds, and the emergence of radicals. These small carbon-enriched particles may aggregate into electrically conductive clusters due to their electrostatic attraction^9^.

Polymer blends physiochemical characteristics and electric conductivity can be improved by adding nanofillers like ZnO nanoparticles in various ratios^10–14^. Parangusan et al.^15^ studied the piezoelectric properties of electrospun nanofibers made from neat polyvinylidene fluoride hexafluoropropylene (PVDF-HFP) and PVDF-HFP/Co-ZnO. It was observed that the tidy PVDF-HFP and PVDF-HFP/2 wt% Co-ZnO nanofibers have dielectric constants of 8 and 38, respectively. These results suggest that the reported nanocomposite can create flexible, wearable, self-powered electrical systems. Thermoplastic polymers such as high-density polyethylene (HDPE) nanocomposite can be reinforced with graphite nanoplatelets, nanodiamonds and carbon nanotube to improvise rheological, thermal and mechanical properties^16–19^. PVC surface modification in the past is carried out using plasma, corona discharge, chemical grafting, electric discharge, metal vapor deposition (MVD), flame treatment or direct chemical modification (oxidation, hydrolysis, etc.), and even some physical modification of the surface. This study aims to increase PVC hydrophilicity in (PVC/HDPE)ZnO that has been irradiated with Ɣ-rays on various media, including water, paraffin wax, silicon oil, and sodium silicate solutions. Gamma irradiation has several advantages over other techniques, including high penetration power, quick processing, uniform-dose distribution, system flexibility, and ability to be used in various environments^20–25^. Gamma irradiation is an eco-friendly method and the most productive^26–31^. This study also aims to increase the hydrophilicity of (PVC/HDPE) for oil/water separation applications. Applications for oil–water separation are crucial for industrial processes like those involving petroleum, metalworking, ship bilge water, and the food industry, which uses fats, oils, and grease, among other things.

Oil/water separation is a crucial subject of study for scientific research and concerns the environment, the economy, and society. On the one hand, the most prevalent pollution in the world today is oily wastewater produced by industries like steel, aluminium, food, textile, leather, petrochemical, and metal finishing. On the other hand, frequent oil leak accidents are extremely concerning because the discharge can cause significant energy loss and catastrophic environmental degradation. Additionally, since even a small amount of fuel oil could jeopardise transportation safety, expelling water from fuel oil is essential in the vehicle, ship, and aviation industries. In response to these enormous obstacles, scientists have consistently focused on creating new methods and materials for oil/water separation. Many common separation technologies are used today, including gravity separation, centrifugation, ultrasonic separation, air flotation, electric field, coagulation, and biological treatment. These methods can handle most separation requirements by carefully integrating physical, chemical, and biological techniques. Due to its superhydrophobicity, high surface area, chemical inertness, low density, recyclability, and selectivity, carbon nanotubes based on membranes have been developed for oil spill clean-ups by Parangusan et al.^32^. The addition of carbon nanotube greatly improves the electrical conductivity of polymer composites with a sudden transition from an insulator to a semiconductor due to the decrease of tunneling distance^33–35^.

Preparing highly dispersed polymer-based nanocomposites made of PVC/HDPE embedded with ZnO nanoparticle using gamma irradiation is one of the primary objectives of the current study. Investigations have been done on how gamma radiation affects the physiochemical characteristics of ZnO nanoparticles. To meet the emergency response needs of effective oil spill separation, it was also examined how to increase the hydrophobicity of blended surfaces by irradiating samples at various media (water, paraffin wax, silicon oil, and sodium silicate solutions). The novelty of current research is the developed nanocomposite blend of (PVC/conductivity HDPE)/ZnO properties for acting as dual functions as oil removal and uniform the electric field distribution in medium voltage cables by surface modification.

## Experimental

### Materials

polyvinyl chloride (PVC) was supplied from Misrelhegaz company, Egypt and high-density polyethylene (HDPE) was supplied from Sabic company, Saudi Arabia with technical data out line in Table 1 from the market and used without further purification. Chemicals such as sodium silicate solutions (Na_2_O_x_SiO_2_) M.W 184–254, paraffin wax (C_n_H_2n+2_ melting point: 50–57 °C) and silicon oil (Xiameter PMX-200 Silicone) were supplied from the market and used as it is.

**Table 1:** Basic physical properties of PVC and HDPE raw materials.

Materials	Chemical formula	Catalog number	Density (specific gravity)
PVC	(H_2_C–CHCl)_n_	Sabic-B1054	Max.1.4
HDPE	(C_2_H_4_)n	–	0.954

### Preparation of (PVC/HDPE)/ZnO by melt extruder methods

Polyvinyl chloride was melt-mixed with HDPE in the ratio (30/70) wt/wt% using a twin screw extruder (CTW100P; Haake Poly lab Rheomix, Germany). The content of ZnO was 5wt% and adding in the meld blends of PVC and HDPE and the rotating screw speed in the extruder was 120 rpm. The extrudates obtained from the twin screw extruder were two-roll milled (Lab Tech Engineering Co., Bangkok, Thailand) at 170 °C for 7 min before compression-molding with a hot press (Lab Tech Engineering Co., Bangkok, Thailand) at 170 °C with a pressure of 150 kg/cm^2^ for 4 min. The moulded (PVC/HDPE)/ZnO composite was cut into test pieces for further experimental evaluation.

### Preparation of ZnO nanoparticles

A typical experiment created ZnO nanoparticles using the traditional sol–gel process. The solution, A of zing salt, was created by dissolving 20.196 g (0.10 mol) of zinc acetate in 600 mL of water/ethanol in a ratio 80/20 v/v% and stirring it for 60 min at room temperature. A solution of 0.20 mol of oxalic acid dehydrates was obtained by dissolving 2.520 g in 800 mL of water/ethanol in ratio 80/20 v/v% and stirred at a temperature of 50 °C for 60 min to create Solution B. Warm solution A was constantly mixed for one hour while solution B was added dropwise. A white sol was obtained, aged to create a gel, and then dried for 24 h at 100 °C. ZnO was finally produced using thermal processing at calcination temperatures of 600 °C for 3 h.

### Gamma irradiation-induced Surface modification of (PVC/HDPE)/ZnO samples

Another advantage of radiation-induced surface modification in four different media (silicon oil, sodium silicate paraffin wax and water) is that it enables tailored modifications ranging from surface to bulk of backbone polymers, unlike photo- and plasma initiation, which imparts surface modification only. The modification process can improve the hydrophilicity or hydrophobicity of (PVC/HDPE)/ZnO samples or their conductivity or modify its oil adsorption. Sheets of irradiated (PVC/HDPE)/ZnO samples are cut into strip samples and re-irradiated with γ rays at 25 kGy in four different media (silicon oil, sodium silicate paraffin wax and water). The irradiation process is carried out under ambient conditions and a dose rate of 0.67 kGy/h is maintained by using a Co-60 source (Irradiation is performed NCRR, AEAE).

### Characterization

An ultraviolet (UV)-visible spectrophotometer (Ultraviolet-3600, Schimadzu) was used to monitor the characterization of the band gab of synthesized ZnO and irradiated ZnO at a scanning range of 200–500 nm. The Fourier-transform infrared spectroscopy (FTIR/ATR) (ATR–FTIR) Vertex 70, Bruker Optik GmbH, Ettlingen, Germany is a technique used to obtain the chemical change structure of surface modified (PVC/HDPE)/ZnO nanocomposite. The powder X-ray diffraction (XRD) technique used the XRD-7000 (Schimadzu, Germany). The process for the crystalline analysis of the ZnO with Cukα radiation (λ = 1.5418 Å). The mechanical properties and tensile characteristics in the dumbbells shape of obtained modified (PVC/HDPE)ZnO samples were measured using an Intron mechanical testing machine under ASTM D638 (model 5569). The surface morphology of (PVC/HDPE)ZnO sample was examined using a Scanning Electron Microscope (SEM) of ZEISS EVO 15 SEM, UK. The contact angle of of synthesized ZnO and irradiated ZnO was measured on a horizontal surface by a KRÜSS EasyDrop DSA20 instrument. AC conductivity σ_AC_(ω) was measured by LCR bridge model Hioki 3532 which was used to measure the impedance Z and the phase angle between the applied AC voltage and the resulting current in the samples of sample (PVC/HDPE)/ZnO and surface modified (PVC/HDPE)/ZnO. The frequency ranged from 0.00 to 500 Hz. The variation in AC conductivity with a frequency at ambient temperature on (ln-ln) scale. The impedance Z, sample capacitance Cp, and loss tangent Tanδ were measured using a programmable automatic 3532 LCR meter. The resistance R was parallel to all capacitance values Cp was taken from the bridge's screen. The module of COMSOL multiphysics software of V5.2 in distribution systems is used underground medium voltage cable (XLPE). The working layer in the analysis test is copper conductor with 3.5 mm radius, inner semiconductor 4.5 mm, XLPE insulation 10.5 mm and outer semiconductor 11.25 mm. All radii have been estimated from the middle of the conductor of copper. The physics properties: AC/DC copper using finite element method, mesh processing: Finer and computer RAM: 8 GB. The relative tolerance in the solution processing in COMSOL software is 2.147 for (PVC/HDPE)/ZnO at 0 kGy and 2.22 at 25 kGy.

## Results and discussion

### Effect of gamma irradiation on AC conductivity, contact angle, structural and optical properties of ZnO nanoparticles

**Figure 1:**
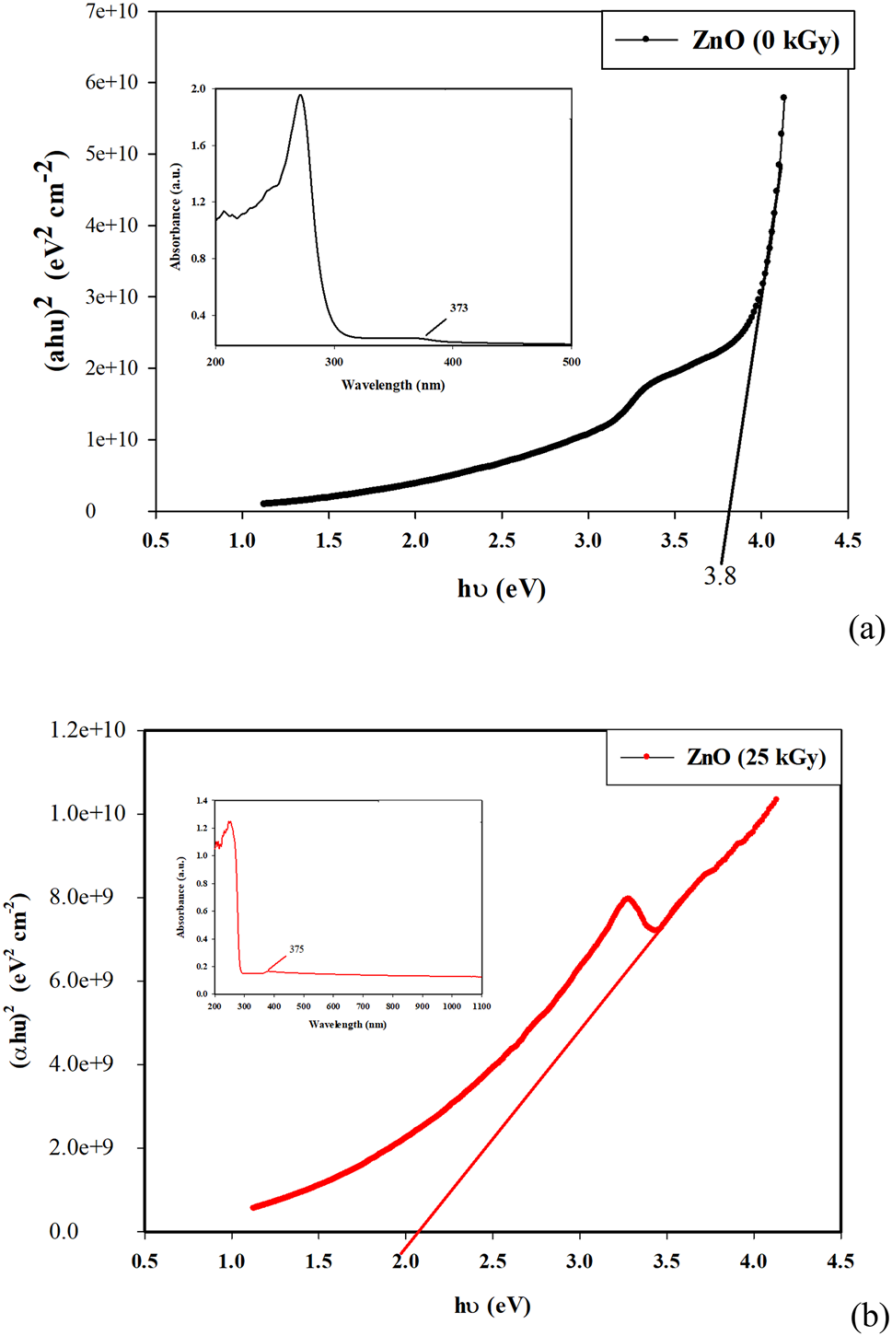
The optical properties of unirradiated and irradiated ZnO NPs at dose of (**a**) 0 kGy and (**b**) 25 kGy.

The optical properties of nanoparticles powder of two samples of unirradiated and irradiated ZnO at dose of 25 kGy were represented in Fig. 1. The Vis–UV spectrum shows characteristic absorption peaks of ZnO (0 kGy) at the wavelength of 242 nm, 271 nm and 373 nm which can be attributed to the intrinsic band-gap absorption of ZnO–NPs because of the electron transitions from the valence band to the conduction band (O_2_ → Zn_3_d)^36^, The absorption spectrum of ZnO–NPs also suggested the narrow nano size particle distribution. The band gap energy of ZnO NPs can be estimated according to the formula

$${\text{E}} = {\text{hc}}/\lambda ,$$

where plank’s constant (h) is (6.626 × 10^−34^ J s), the velocity of light (c) is (3 × 10^8^ m s^−1^) and λ (373 nm) is the wavelength. The band gap energy of ZnO NPs was calculated as 3.8 eV.

After an irradiation dose of 25 kGy, the characteristic absorption peak of the ZnO NPs at wavelength of 373 nm was slightly red-shifted relative to the absorption maximum of 375 nm. It may be due to the defect of crystal structure by the oxygen vacancy during gamma irradiations. This growth of the defect site leads to a decrease in energy gaps to become 2.08 from 3.8 eV with an irradiation dose of 25 kGy. The development of the defect state could be caused by the reduction of compressive stress in the ZnO films. The reduced compressive stress of irradiated ZnO may be attributed to the well-aligned ZnO hexagonal nanoparticles^37^.

**Figure 2:**
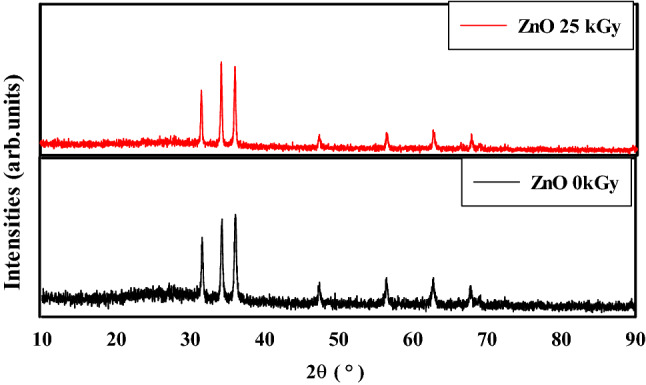
The XRD analysis of un-irradiated and irradiated ZnO nanoparticles at dose of 25 kGy.

Figure 2 shows the XRD analysis of un-irradiated and irradiated ZnO nanoparticles at 25 kGy. The XRD curve shows 7 intensity peaks located at 2*θ* = 31.57°, 34.13°, 36.00°, 47.57°, 56.45°, 62.72° and 67.70°, confirming the hexagonal wurtzite structure of ZnO NPs according to^38^. After gamma irradiation the 2θ of 31.57°, 34.13°, 36.00° were shifted to 31.66°, 34.31°, 36.15°. Also the d-spacing was decreased from (2.831, 2.617, 2.489) Å to (2.823, 2.611 and 2.482) Å, respectively. The decrease of d-spacing after gamma irradiation is expected due to metal oxide defect and atom displacement phenomena^39,40^.

**Figure 3:**
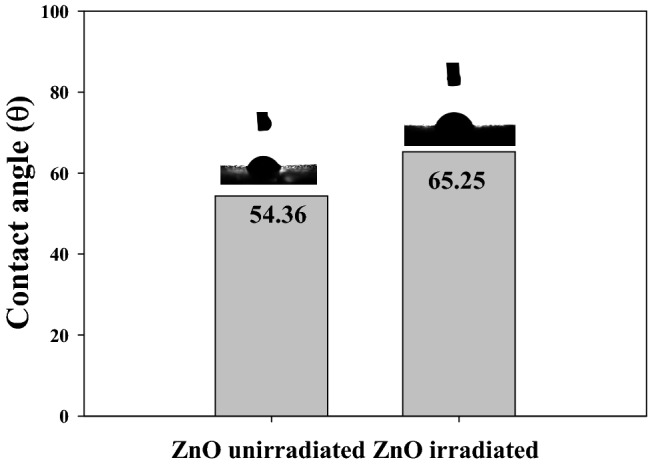
Show the contact angle of un-irradiated and irradiated ZnO nanoparticles at dose of 25 kGy.

The effect of gamma irradiation at dose of 25 kGy on the contact angles of ZnO was investigated. Figure 3 shows the wettability character of un-irradiated and irradiated ZnO nanoparticles by measuring the contact angles of the water drop. The contact angle of the irradiated sample is increased from 54.36° to 65.25° compared to the blank sample. The increased contact angle of the irradiated sample is due to the increased ZnO orientation and the well-aligned ZnO hexagonal nanoparticles, as confirmed by band gab data analysis and XRD data analysis.

**Figure 4:**
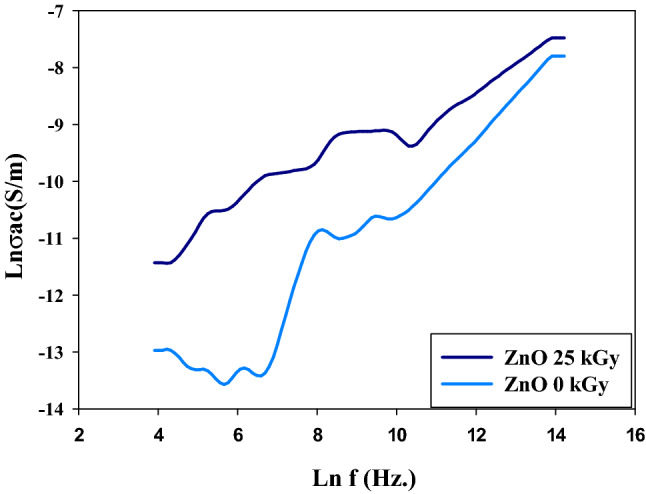
Shows the electric AC conductivity of un-irradiated and irradiated ZnO nanoparticles at 25 kGy.

Figure 4 shows the variation in AC conductivity as a function of ln frequency at two irradiation doses (0 to 25) kGy for ZnO nanoparticles. It is observed that the two samples exhibit different AC conductivity phases with different values over the whole range of measured frequency. The Ac conductivity of the irradiated sample is higher than unirradiated samples. The possible increase in defects on ZnO’s crystal structure and the expansion of conduction routes between ZnO particles are linked to the growth in AC conductivity. The bulk conductivity may rise due to more charge carriers being able to “jump” by tunneling, and it also rises with gamma irradiation exposed^41,42^. Table 2 outline the main change in the physiochemical properties of irradiated and unirradiated ZnO sample.

**Table 2:** The main change in physiochemical properties of irradiated and unirradiated ZnO sample.

Parameter	ZnO (0 kGy)	ZnO (25 kGy)
λ_Max_ (nm)	373	375
Band gab (eV)	2.08	3.8
Contact angle	54.36°	65.25°

### Morphology of (PVC/HDPE)ZnO nanocomposites

**Figure 5:**
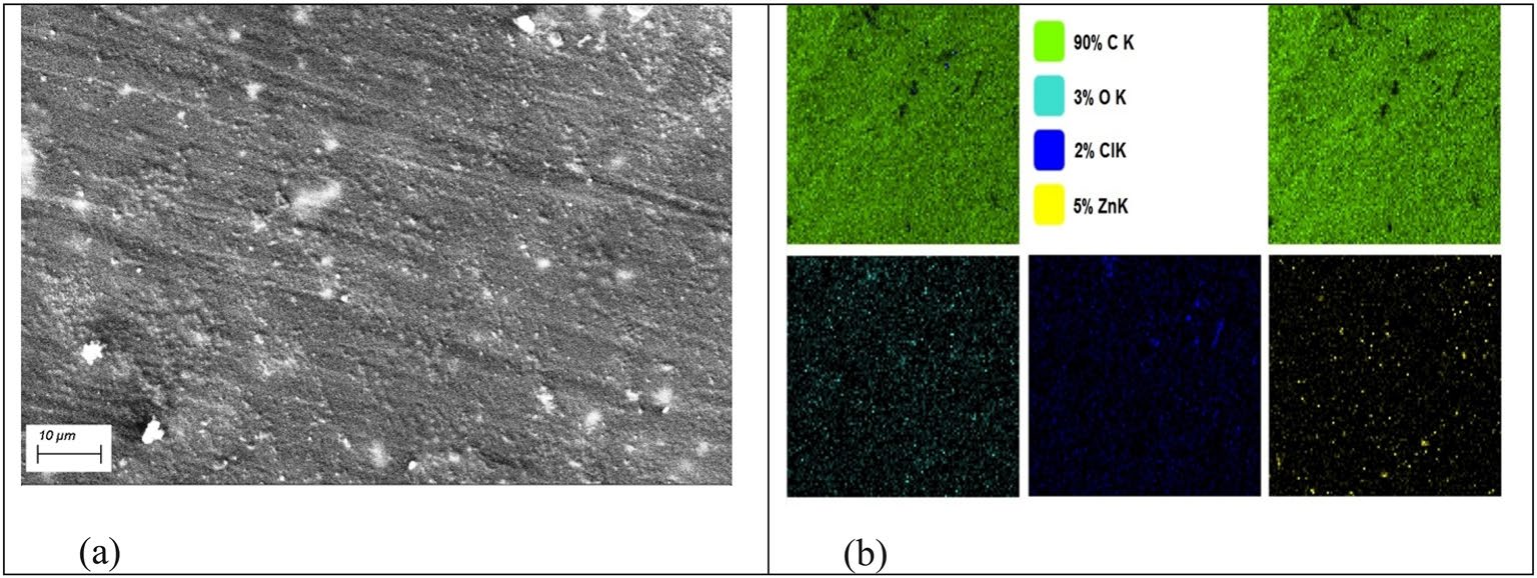
SEM (**a**) and EDX/mapping (**b**) of (PVC/HDPE)ZnO nanocomposite.

SEM images of 5% ZnO and PVC/HDPE mixture are shown in Fig. 5a. It is quite clear that the addition of ZnO nanocomposite caused the surface of the sample to appear rougher. This is useful in increasing oil adsorption on the sample’s surface. The same result agreed with the results obtained by Dai et al., Haq et al. and Barroso-Solares et al. Dai et al.^43^ found that the surface roughness of poly(lactic acid) (PLA) fibers can be controlled by increasing the content of Zn^2+^ in metal framework of (ZIF-8). Oil adsorption is accelerating due to the higher roughness of (PLA/ZIF-8) fiber surface. Haq et al.^44^ prepared oil adsorbent resin system includes (unsaturated polyester + epoxidized soybean oil + nanoclay) with varying nanoclay amounts. He and coauthor obviously that the surface’s roughness is increased due to the addition of nanoclay. Barroso-Solares et al.^45^ prepared mixed nano fibers of two polymers, poly(methyl methacrylate) (PMMA) and polycaprolactone (PCL), with weight ratios of 70/30, 50/50 and 30/70 incorporated with 1 wt% of silicon oxide fumed nanoparticles. It was noted that the 50/50 PMMA/PCL sample shows the most promising compromise between the oil absorption capacity, oil selectivity, and mechanical properties, this is due to the increased roughness of the 50/50 PMMA/PCL fibers respect to the other two samples (70/30 and 30/70). Figure 5b shows better evidence regarding the distribution of ZnO nanoparticles obtained via elemental EDX mapping of Zn and O as the main elements from ZnO and of C and Cl from (PVC/HDPE) blend in the case of (PVC/HDPE) ZnO nanocomposites. These two elements of (Zn and O) are present in the surface in well distribution identified in PVC/HDPE matrix. EDX / mapping analysis did not provide evidence of any other trace elements.

### The surface hydrophobicity modification of (PVC/HDPE)/ZnO samples

**Figure 6:**
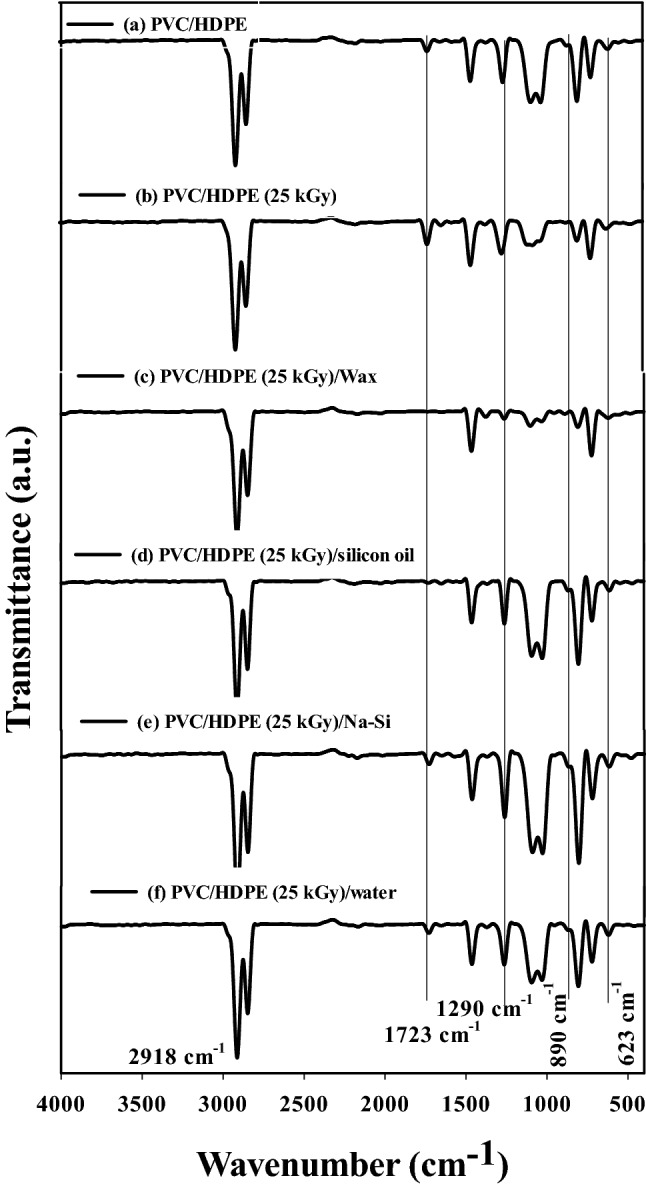
The FTIR spectra of PVC/HDPE (**a**) unirradiated, (**b**) irradiated 25 kGy, (**c**) in paraffin wax, (**d**) in silicon oil, (**e**) in sodium silicate and (**f**) in water.

The obtained compatible blends of (PVC/HDPE) ZnO irradiated at different media (paraffin wax, silicon oil, sodium silicate and water) as an efficient method for the hydrophobicity modification of the surface of blends to use in oil/water separation. Surface modification is modifying the surface of (PVC/HDPE) ZnO blends by bringing chemical and physical properties different from the blank sample. The physicochemical properties of surface-modified (PVC/HDPE) ZnO blends were performed using other characteristics such as FTIR, mechanical properties and XRD. The chemical structure of the surface modification of (PVC/HDPE)ZnO blend films using FTIR spectroscopy is shown in Fig. 6. The two FTIR peaks located at 2918 cm^−1^ and 623 cm^−1^ are characteristic peaks of C–H and C–Cl bonds of PVC molecules, respectively. The peak at 1290 cm^−1^ is attributed to trans CH_3_ in polyethylene molecules^46^. The peak at 1723 cm^−1^ attributed to C=O groups was observed in unirradiated (PVC/HDPE)ZnO samples and irradiated samples at sodium silicate and water media. On the other hand, the C=O groups completely disappeared in case (PVC/HDPE) ZnO samples were irradiated in paraffin wax and silicon oil. The peak intensity of 1290 cm^−1^ was increased in the case of the irradiated sample at silicon oil and sodium silicate media due to the formation of Si-CH_3_ at 1250 cm^−1^^47–49^.

**Figure 7:**
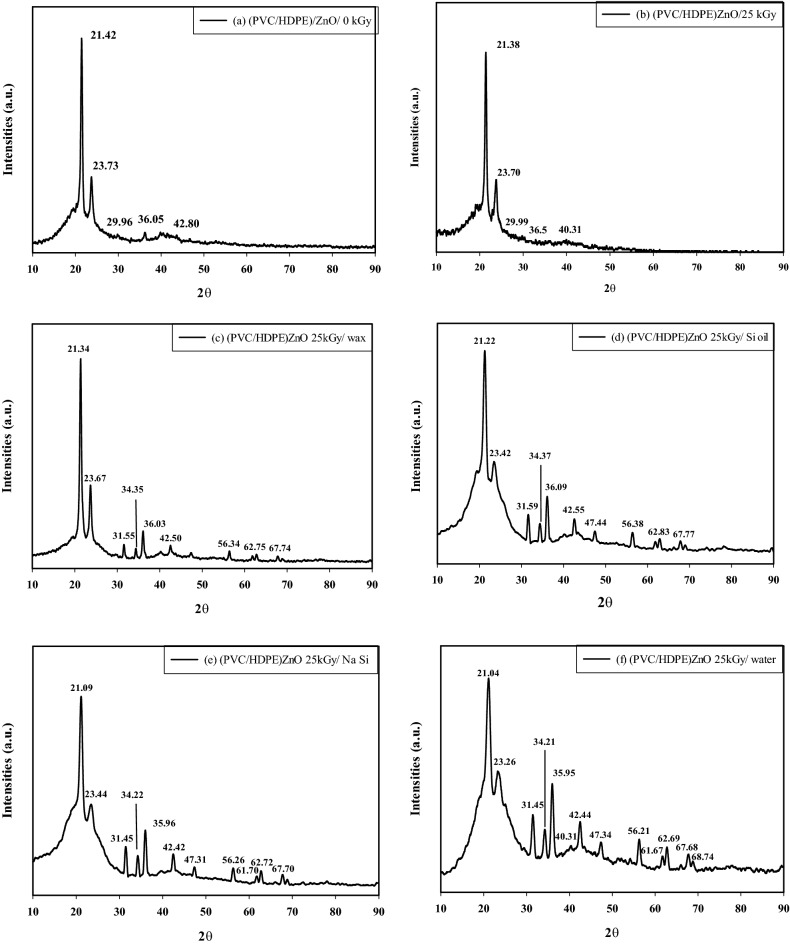
The XRD spectra (**a**) unirradiated, (**b**) irradiated (25 kGy), (**c**) in paraffin wax, (**d**) silicon oil, (**e**) sodium silicate and (**f**) water of (PVC/HDPE)ZnO blends.

Figure 7 shows the X-ray diffraction (XRD) patterns of the blank and the modified surface of irradiated (PVC/HDPE) at the dose of 25 kGy. It is noted that, the XRD peaks of ZnO is not clear in Fig. 7a,b samples of (PVC/HDPE) at the dose of 0 kGy and 25 kGy, respectively due to the surface modification of (PVC/HDPE)/ZnO leading to concentration the nanoparticles in the surface of plastic sheet similar to the bulk of the samples in Fig. 7c–f. The characteristic XRD peaks of HDPE are located at ~ 21° and 23°, which correspond to the typical semi-crystalline nature of orthorhombic unit cells of (110) and (200) reflection planes, respectively^50–52^. Figure 7 confirms plane (110)’s diffraction peaks are almost changing in all modified (PVC/HDPE) samples. This means the modified surface is placed on the plane (110) of HDPE^53^. Also, Fig. 7 shows a very broad XRD peak of PVC ranging from 14° to 24°; this indicates that PVC is amorphous^54^. Also, XRD in Fig. 7 represents the XRD pattern for ZnO nanoparticles located at 2∂ = 31.45°, 34.22°, 35.96°, 42.42°, 47.31°, 56.26°, 62.72°, 67.70°, 67.84°, 68.96° and 72.45° correspond to reflections from the crystal planes 100, 002, 101, 102, 110, 103, 200, 112, 201, 004 and 202 This ensures the formation of the ZnO in a pure hexagonal wurtzite phase^38^.

**Figure 8:**
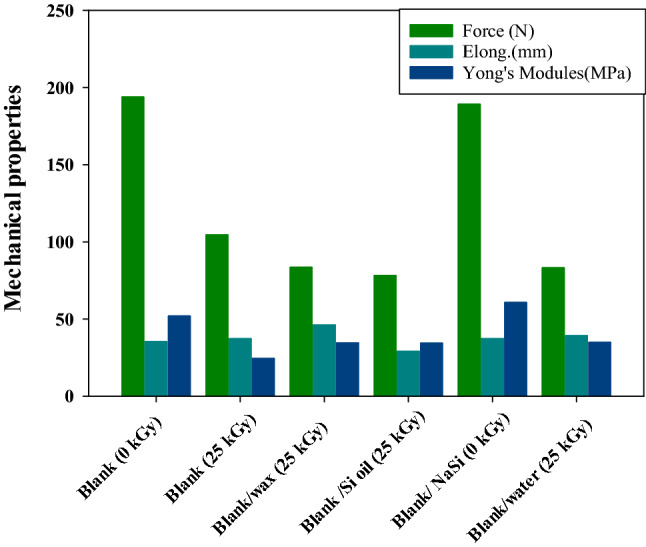
Comparison of mechanical properties regarding Force, Elongation and Young’s modulus.

From the bar graph Fig. 8, it can be perceived that the force (N), elongation (mm) and Young’s modules (MPa) of unirradiated (PVC/HDPE)ZnO blends are much more than all irradiated (PVC/HDPE)ZnO blends samples. The Young’s modulus and force of blank (PVC/HDPE)ZnO (0 kGy) and at (25 kGy) were 51.7 MPa, 193.66 N and 24.22 MPa, 104.3 N, respectively. After gamma irradiation, Young’s modulus and force were observed to be decreased due to the defect state of the atomic bonds in blends induced by gamma irradiation. On the other hand, Young’s modulus and force were observed to be decreased in all treated samples after surface treatment. These results justify that the surface modification contributed to changes in the surface’s chemical structure; falls in mechanical properties can be due to the low interfacial bonding between the bulk and surface of the blend chain. The intense interfacial bonding between the bulk and surface of the treated blend chain may cause an elongation increase compared to blank samples. Further, the mechanical properties of the (PVC/HDPE)ZnO blend gamma irradiation treated in Na–Si exhibit higher Young’s modulus and force value than other treated (PVC/HDPE)ZnO blend samples. This may be attributed to a harder physical bonding effect and the formation of weak bonds on the (PVC/HDPE)ZnO blends surface.

### Effects of gamma irradiation and surface modifications on the removal of oil

**Figure 9:**
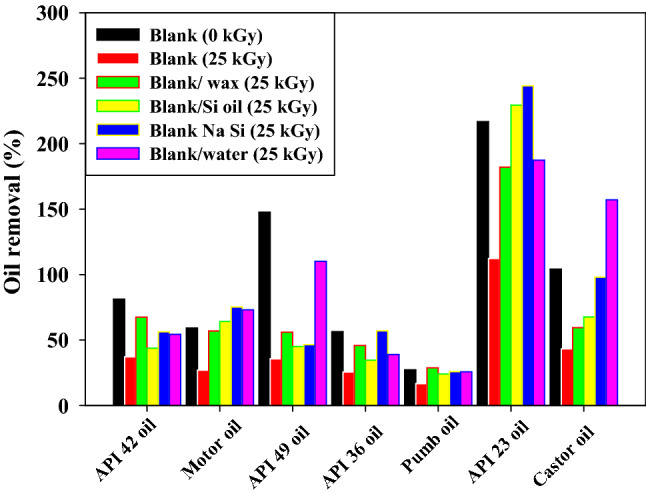
The adsorption capacity of six different modified irradiated (PVC/HDPE)ZnO composites for removal of six kinds of oils.

Oils absorb on surfaces due to a surface phenomenon. As a result, adsorbents have vast surface areas, most of which are interior surfaces that enclose the massive pores and capillaries of highly porous materials. As shown in Fig. 9 the oil-adsorbed power of (PVC/HDPE)/ZnO is decreased after irradiation. One of the most reasons is the porous adsorbents become smaller and reduce their capability for efficiently adsorbing due to gamma irradiation-induced cross-linked reactions. While after surface modification, the four modified (PVC/HDPE)/ZnO show a wide spectrum of adsorption capability for the six different kinds of oils. The performance characteristics of adsorbents largely relate to their intraparticle properties such as surface chemical compositions and surface hydrophobicity. The hydrophobic surface area of modified samples and the functionalised distribution of groups concerning pore size and roughness surface generally are primary determinants of adsorption capacity.

On the other hand, the density of removal oil and API number (American Petroleum Institute number is used to identify the oil and gas wells) should be considered. The effect of API on the adsorption potential of oil was in inverse relations ship. As show in Fig. 9 the percentage adsorption capacity of oils was observed to increase in direct proportion with a decrease in API value and at an inverse proportionality with low functionalized sites in modified (PVC/HDPE)ZnO samples due to the presence of functional groups found in crude oils^55^. According to previous literature, crude oils have other groups containing –C=C–, C=O and OH with excess sulfur and nitrogen^56^. For castor oil, the adsorbent sample of (PVC/HDPE)/ZnO irradiated at a 25 kGy in water media increased from 73 to 157% compared to the blank sample irradiated at 25 kGy. As depicted in Fig. 9, the adsorption uptake was observed to be changed depending on the kind of adsorbent. As the functionalized sites on the surface were increased from irradiated samples at 25 kGy in water and NaSi solutions, the adsorption uptake of oil increased consistently. The adsorption uptake of motor oil increased from 26%, 56%, 64%, 73% and 75% for modified irradiated samples in air, wax, Si oil, water and NaSi. The highest adsorption capability of oils was observed in samples functionalized by C=O groups. The availability of the functional group sites on the adsorbed surface enhanced the oil removal capability. The results suggest that the surface characterization of the (PVC/HDPE)/ZnO can be modified to enhance the oil adsorption potential.

### AC conductivity of surface modified (PVC/HDPE)/ZnO

**Figure 10:**
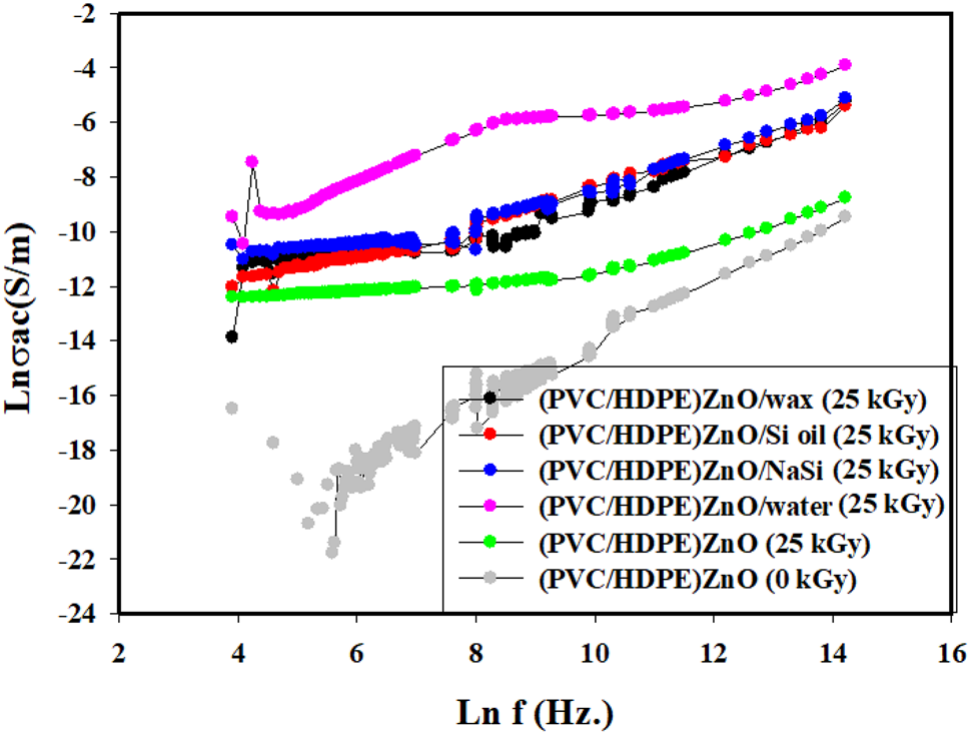
The AC conductivity of surface modified (PVC/HDPE)/ZnO.

Figure 10 shows the AC conductivity of (PVC/HDPE)/ZnO, which varied in the same behavior independently on the surface modification and the type of function group formation. However, it should be noted that each sample’s percolation threshold depends on the gamma irradiation condition in different solutions. Figure 10 shows the lowest conductivity of un-irradiated samples of (PVC/HDPE)/ZnO seemed to increase twice after irradiation at a 25 kGy. This is due to the gamma irradiation-induced defect by atom displacement that increased the electron transition after the formation of holes. Additionally, dehydrochlorination of PVC molecules brought on by gamma irradiation may result in the formation of conjugated double bonds. Throughout these conjugations, the electrons then become mobile. Consequently, electron mobility contributes to the material’s overall conductivity^57,58^.

On the other hand, the two irradiated samples of (PVC/HDPE)/ZnO in water and NaSi solutions had higher conductivity values for the same given frequency. This is due to forming new function groups in the modified surface detected by FTIR data. The functional groups can serve as a host matrix for electron transition.

### Simulation and modeling of electric field distribution inside irradiated (PVC/HDPE)-ZnO) nanocomposite at the dose of 25 kGy

**Figure 11:**
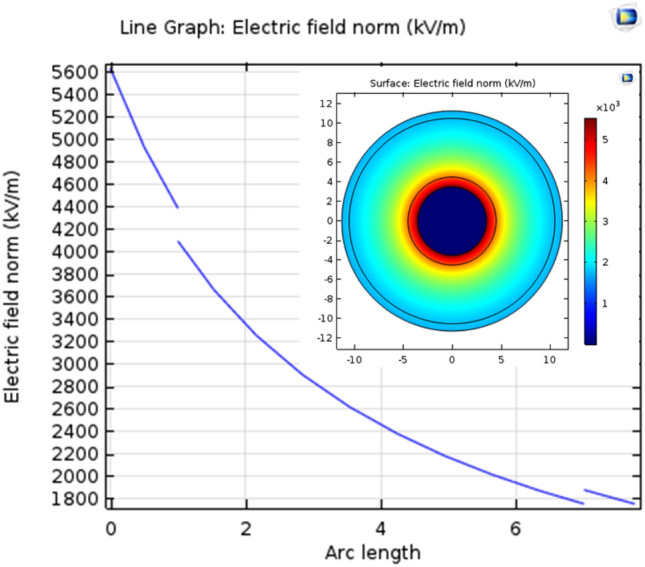
Electric field distribution in medium-voltage of unirradiated (PVC/HDPE)ZnO.

**Figure 12:**
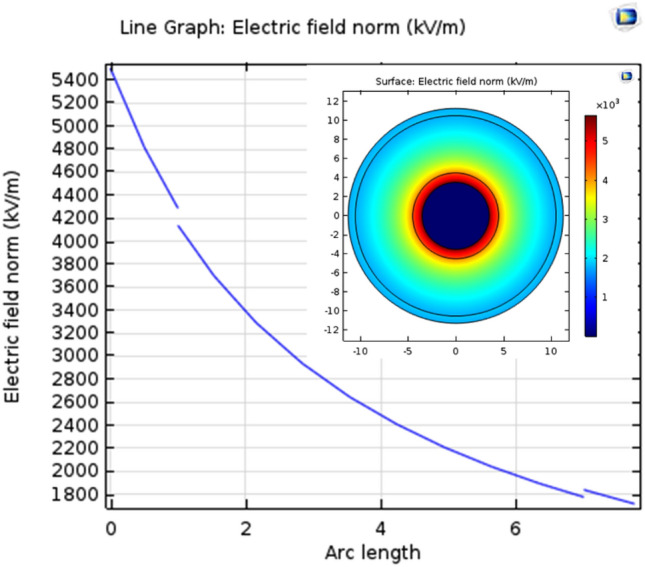
Electric field distribution in medium-voltage cables of irradiated (PVC/HDPE)ZnO water.

To simulate the distribution of the electric field in MV cables, utilise COMSOL Multiphysics. The distribution of the electric fields inside the (PVC/HDPE)ZnO unirradiated sample is depicted in Fig. 11. The electric field distribution inside the sample is not uniform at 1 mm of arc length. The electric field distribution for irradiation (PVC/HDPE)ZnO/water is becoming uniform and steadily declines from the interior to the outside, as shown in Fig. 12. This is due to the new C=O function groups ability to maintain an even electrical field while lowering electrostatic tension.

## Conclusions

- ZnO was irradiated at dose of 25 kGy for improved it physiochemical properties, The Vis–UV spectrum of ZnO shows an absorption peak at a wavelength of 373 nm that was slightly red-shifted to 375 nm for an irradiated sample of ZnO at a dose of 25 kGy due to the defect of crystal structure by the oxygen vacancy during gamma irradiations.
- This growth of the defect site leads to a decrease in energy gaps from 3.8 to become 2.08 eV.
- The contact angle of the irradiated sample is increased from 54.36° to 65.25° compared to the blank sample.
- Surface modification of the (PVC/HDPE)ZnO with gamma irradiation treatment was investigated.
- The gamma irradiation process at dose of 25 kGy was performance in four different media such as (paraffin wax, Si oil, NaSi and water) to improve the surface by a functionalized group of PVC/HDPE)ZnO samples.
- FTIR spectra revealed chemical change on the (PVC/HDPE)ZnO surface after gamma irradiation treatment in water and NaSi.
- The C=O peaks appeared in the expected FTIR, confirming the sample surface's alteration.
- The surface of the (PVC/HDPE)ZnO sample enhances its oil removal capability compared to blank samples.
- The adsorption uptake of motor oil increased from 26%, 56%, 64%, 73% and 75% for modified irradiated samples in air, wax, Si oil, water and NaSi.
- The highest adsorption capability of oils was observed in samples functionalized by C=O groups.
- According to COMSOL Multiphysics, the irradiated (PVC/HDPE)ZnO sample in water shows uniform electric field distribution in medium voltage cable (22.000 v).
- The future work of surface modified (PVC/HDPE)ZnO sample can be expanded the use of (PVC/HDPE)ZnO based on their AC conductivity that was an enhancement.

## Data Availability

The data that support the findings of this study are available from the corresponding author upon reasonable request.
